# Exploring States of Panacea and Perfidy of Family and Community Volunteerism in Palliative Care Giving in Kanye CHBC Program, Botswana

**DOI:** 10.4103/0973-1075.63129

**Published:** 2010

**Authors:** Simon Kangethe

**Affiliations:** Department of Bachelor of Business Administration (BBA), Centre for Continuous Education (CCE), University of Botswana, Botswana

**Keywords:** Community home based care program, HIV/AIDS, HIV/AIDs clients, Palliative caregivers, Panacea, Perfidy, Volunteerism

## Abstract

**Aim:**

The study aims to explore the attitudes and perceptions of family and community palliative care givers pertaining to volunteerism.

**Objective:**

The main objective is to involve palliative caregivers and their supervisors in assessing their contribution to care and evaluate their state of volunteerism.

**Materials and Methods:**

The study attracted qualitative design and involved 82 palliative caregivers in 10 focus group discussions; one-to-one interviews with the nurses supervising them. Two slightly different interview guides were used as research instruments.

**Results:**

Findings indicate that palliative care giving volunteerism is motivated and sustained by: (1) Principles of love emanating from blood and kinship relations; (2) Patriotism and community responsibility over one another; (3) Adherence and respect of their culture and government call. Volunteerism was also found challenged by: (1) Predominance of the elderly and lowly educated women; (2) Poverty and heavy caseload; (3) Being shunned by the youth; (4) And lack of morale, recognition and motivation.

**Recommendations:**

The study recommends: (1) Socializing boys early enough in life into care giving; (2) Offering incentives to the caregivers; (3) Use of public forums to persuade men to accept helping women in carrying out care giving duties; (4) And enlisting support of all leaders to advocate for men’s involvement in care giving.

## INTRODUCTION

Palliative caregivers refer to informal family and community individuals who stay with CHBC clients (HIV/AIDS and other chronically ill persons) to offer palliative care.

Palliative care is the care that is carried out, not to heal, but to relieve pain, distress, psychological loss, feeling of worthlessness and anxiety, and gives hope, dignity, esteem, happiness, and inculcate the feelings that all will be well to a sick person.

Perfidy is a bad and undesirable state of affairs.

Panacea is a desired state of satisfaction, motivation and high esteem leading to psychological, emotional, physical and social well being.

Current debates of national, regional and international magnitude seem to suggest the importance of volunteerism to foster development tempo especially in areas with low levels of qualified human resource.[1,2] The United Nation’s Volunteer Unit has been a force to reckon with in encouraging volunteerism by both the qualified personnel and ordinary persons. It has, therefore, attracted the researcher to look into the attitudes, thoughts and perspectives of the palliative volunteer caregivers to inform the existing levels of volunteerism, the drive in the present palliative care giving in Kanye CHBC program, and investigate whether such volunteerism was sustainable. In this era of HIV/AIDS, countries like Botswana, seriously challenged by the HIV/AIDS pandemic, are desperate for the roles of the volunteers to do care giving of persons living with HIV/AIDS and other terminal illnesses. This is because of inadequate trained staff to arrest the situation.

The threat of the dwindling spirit of volunteerism[3] is another worrying issue that has prompted a research analysis of palliative care volunteerism as practiced and perceived by the palliative caregivers themselves who are the main stakeholders in volunteering to care for HIV/AIDS and chronically ill clients. Perhaps the findings could put volunteerism into its proper position and change the dwindling position to take a more productive path. There have been indications from several research undertakings that care giving under the current volunteerism spirit in Botswana has had several challenges and therefore negatively impacting on the quality of care discharged. Assessing the situation on the ground was found critical.[3–5]

While it is an incontrovertible fact that the country saves a lot of money when people living with HIV/AIDS and other chronically ill persons are taken care of at home where clinical and palliative services are offered by both the family and community caregivers, it is also worth taking account of the enormous value of love, compassion, concern, security, esteem, assertiveness that these clients receive from family and community caregivers.[4,6–10]

The volunteerism of this nature, with deep social capital and love embedded in people’s hearts is a social panacea that has far reaching intrinsic and extrinsic values to care giving. Not withstanding the efficacy and effectiveness of these important volunteer service providers especially in resource strapped countries of the developing world, palliative care to the clients with chronically and debilitating diseases is especially critical so that proper management of the diseases can be achieved.[11]

This invaluable support and contribution has complemented and augmented government efforts in a huge way. Such gestures could, in an immense way, make not only the national priorities and policies a reality as embedded in Vision 2016 and other important documents such as National Strategic frameworks on HIV/AIDS (2003-2009), National Development (NDP) 9 and 10, but also contribute to the country’s international efforts in meeting the Millennium Development Goals (MDG) of reducing the HIV/AIDS and affording the citizens a healthy life. The act will also contribute immensely to the efforts of the universal access to appreciable state of health especially by the year 2015.[12–15]

Family and community palliative caregivers perform a variety of roles that help people with HIV/AIDS adhere to treatment regimens, relieve pain, psychological loss, loneliness, anxiety, feeling of worthlessness, avoid unnecessary hospital admissions, reduce reliance on formal caregivers, remain at home longer, and maintain good quality of life.[7,11,16] Traditionally, family members have served as the primary caregivers for seriously ill individuals. Because HIV care involves more diverse social networks, many HIV-positive individuals have redefined family boundaries to include lovers, friends, and other not so distant relatives. This scenario is especially applicable in most developing countries where kinship ties are still not breaking at a high pace as modernization, westernization, eurocentricism and globalization take toll.[4,17]

Though palliative and clinical care needs the appropriate and specific resources to handle chronically ill persons, this is not always so as dearth of health services have challenged many countries of the developing world.[9] This has necessitated that the family and community caregivers, usually in community care programs, irrespective of their shortcomings such as age and illiteracy, be on call to handle forceps to roll the bandage, nurse the wounds, and administer various pain killing medications as usually directed by the health practitioner. The presence of a qualified health practitioner though absolutely necessary in home care health settings, may not be forthcoming as clinics and hospitals crowd with more urgent clinical and palliative cases. The home-based care concept, then, has to offer the answer. It is the understanding of this environment and the need to exploit and maximize community goodwill and human capital from its citizenry that the government of Botswana institutionalized community home based care programs and integrated them within the existing mainstream health care system.[4,10,18–20]

## MATERIALS AND METHODS

### Research design

Qualitative design was used in the study seeking to explore the thinking, perceptions, feelings and attitudes of the palliative caregivers on their contribution to care giving and exploring other challenges such as the challenges inherent in the care giving volunteerism. According to Neuman,[21] qualitative research is concerned with meaning, that is, how people make sense of their lives, and experiences

### Sampling selection procedure

All the 140 registered primary palliative caregivers, as they appeared in the CHBC register, were conveniently picked for study inclusion with 82 (59%) of them turning up for ten focus group discussions. Also, all the four CHBC nurses and their coordinator were conveniently picked to participate in the study. They were picked because of their mandate of supervising the CHBC program and the caregivers

### Research instrument

An interview guide was used to steer 10 focus group discussions (FGDs) with family or primary palliative primary caregiver respondents. In addition, one to one in-depth interviews were conducted with five nurses using the same interview guide that only differed slightly with the one for the palliative primary caregivers. The two sets of information corroborated one another. Nurses formed the caregivers’ supervision team.

### Ethical and legal requirements

The study followed all appropriate legal and ethical issues. The research participants were widely consulted and the study objectives were well described in advance to ensure their informed consent. The researcher in his introductory interaction with the respondents promised them anonymity, treating them with respect and dignity and to maintain confidentiality of their responses.[21] The researcher had complied with all the research permit application procedures from the department of the Research and Development Committee Board (HRDC) and therefore given the study’s research permit.

### Research domain

The study, carried out in December (2005) and January (2006), was conducted in Kanye village, one of the biggest and also the oldest villages in the country with a population of more than 40,000 as at the 2001 census.[22] The village is well endowed with health facilities, boasting five clinics and two health posts, and a bigger Seventh Day Adventist (SDA)-run referral hospital. Though Kanye CHBC program was one of those which by government standards was said to be doing well, high death rates were still being experiencing in the area among the CHBC clients,[23] hence it was felt worthwhile to do the study in the area.

### Data analysis and interpretation

To carry out data analysis, both the information from the focus group discussions with the palliative primary care givers and one to one interviews with the nurses was taped and then transcribed. The crude data was sorted and reduced to manageable categories and themes. Quotes, words, analogies, proverbs were noted, while tables and graphs were used to present the data and therefore infer the findings. There was double translation of the instruments, that is translation from English to Setswana and then from Setswana to English by two independent translators, the two parties coming together to settle on the difference. This helped reduce data and result bias. Concerning the reliability and validation of the information collected, the five nurses answered more or less the same questions as the primary caregivers. The two sets of information crosschecked and corroborated each other.

## RESULTS

### Profile of the caregivers

#### Age, gender and educational dimension of the caregivers

The caregiver respondents’ ages ranged from 18 to 85 years, most of whom were women. While 46 caregivers constituting 56% were aged from 50 and above, 28 or the relatively older caregivers (60 years and above) constituted 34% of the total caregivers. The study revealed that most caregivers especially those above 60 years indicated they were poor, and 36% of them had more than one client. This was a painstaking and overwhelming experience and constituted a heavy caseload. Eighty-eight percent (88%) of the caregivers had no any income to support themselves and they indicated that they were full time volunteers in care giving

“We do nothing except care giving”

Generally, caregivers expressed being overwhelmed as they were getting only little support from other family members, relatives and communities at large. Some caregivers broke into tears as they explained the circumstances of being left alone to do care giving by their families and relatives. Bursting into tears, one had this to say:

*“I cannot get time to go and look for a job as I’m alone caring for my father. They all went for good leaving me to struggle with care giving”*.

Seventy four per cent of the caregivers had either never been to school or had only primary level education. Only five per cent of the caregivers had tertiary education. Illiteracy was found to contribute to low care productivity and poverty. This was psychologically disenabling as most of those who had never been to school were also elderly and said they had challenges of adequately understanding the dynamics of care giving.[4,24] On gender, findings indicate that the program faces serious gender skewed dimension with 80 (98%) being women and only two men (constituting 2%).[4]

### Factors driving palliative care giving

#### Blood and kinship ties

The majority of the caregivers indicated that they provide care to their sick family members because they feel obliged to do so. They indicated that while close kins have an obligation to care for their relatives, a sizeable number felt they had a societal and cultural obligation to take care of the sick. The following quotes indicate and support the success of care giving volunteerism as driven by love emanating from blood relationship and a feeling of community responsibility:

“We have a responsibility to take care of them. They are our children whom we have given birth to and brought up”

“Care giving is difficult. But we have love for them. They are our husbands, parents, children and blood relatives”

“We have accepted and appreciated caring job because we take care of our own communities”

The above quotes indicate that the love ingrained and grounded in people’s hearts is enough to drive care giving volunteerism.

#### National interests and patriotism

Forty-one caregivers (50%) of the caregiver respondents indicated that they supported volunteering to take care of the sick in the communities because the call had been officially made by the government. Caregivers said they understood the load that the government was carrying in this era of HIV/AIDS and therefore honored the call to assist through local leadership structures which they respected. A smaller number, however, indicated that the call for volunteerism was also grounded in national documents like vision 2016 which they embraced and supported.

The following quotes were echoed by many in the discussions:

“We accept volunteerism in care giving because our government has officially asked us for a helping hand”

“We respect and concur with our leaders’ request for us to volunteer to take care of the sick”

#### Love emanating from religious principles

Majority of the palliative primary caregiver respondents indicated that besides care giving volunteerism, being culturally ordained in Setswana culture and many other traditions, it was a religious vocation and a calling which they had embraced. Caregivers, therefore, felt they were doing their religious obligations as is provided for in the holy book of the bible. They also comforted themselves that although they are not paid for the arduous task of care giving, God will take care of them.

“Caregiver volunteers will only be rewarded by God”

“Care giving volunteerism is a show of religious love and obligations to our loved ones”

#### Setswana culture

Sixty one (74%) of the caregivers contended that although palliative care giving volunteerism needed to consider incentives, they felt they were fulfilling their societal and cultural obligations. This is because volunteerism, especially on helping the sick was grounded in Setswana culture. Doing what was culturally ordained and grounded left them with satisfaction of serving both their cultures and their country. However, about a third of them complained that some aspects of culture that are exploitative and retrogressive be left out. The following quotes support their arguments.

“Volunteerism without any incentives is very discouraging these days”

“You cannot volunteer on an empty stomach and be expected to be productive”

“The culture that imposes burden to women to be volunteer caregivers should change”

## DISCUSSION

Care giving quality and the spirit of volunteerism, as the study results suggest, has been affected by the age of the caregivers, their literacy level and having elderly women predominating the care giving work. This has worked against care giving efficiency and quality as the elderly and the aged, despite their experience which is however a positive score or panacea to care giving, find it difficulty to adequately understand the dynamics of care giving especially changes in disease progression. In their research in Botswana, Atta and Fidzani[24] found that over 50% of caregivers in most of the Botswana CHBC programmes are old women who may be challenged to adequately follow the disease progression and its dynamics. Research by Jacques and Stegling[3] in Kweneng, Botswana, found three clients with caregivers who were not able to discharge their roles due to old age or disability. Aging definitely impacts negatively on the quality of care giving and indicates the perfidious state of care giving. If the care giving is to be left to the elderly only, then the family structures providing for care giving could be stretched enormously beyond their capacity with the resultant inadequate productivity and decreased interest in care giving spirit.[25]

Contrastingly, elderly people have their special role in care giving. This is because most of the care business especially in the developing world is handled by them.[9,26] The fact that they are willing to care and by the virtue of the fact that most of them are out of productive occupations is an important factor and panacea that care giving structures have exploited and benefited from. The 2003 United Nation’s Secretary General’s report on women in Southern African countries found that two thirds of care is handled by the elderly.[27]

- It is recommended that changing care giving policies to give room to paid incentives could possibly attract the young, and especially men in the care giving occupation. On the findings that care giving was gender skewed, with women predominating the care field, other studies by Munodawafa[18] found all but one male caregiver in Tutume while in Molepolole, all caregivers were females. This state of affairs has not augured well with feminists who see it as perfidious to care giving and presents a gender exploitation process. This is because women have other domestic chores, with care giving presenting further challenge or state of being overwhelmed. This has also been a strong reason driving women to poverty.[28–30]
- It is recommended that societies need to work and persuade men to co-share the care giving assignments with women as the HIV/AIDS epidemic needs to enlist the support of all in the community. On educational level of the caregivers, other studies showed more or less the same trend and pattern of having a bigger number of palliative caregiver volunteers being either illiterate or semi illiterate. In a study by Phorano, Nthomang and Ngwenya[31] on caregivers in Maun and Kweneng, 33% had lower primary education. This is perfidy to care giving.
- It is recommended that on - the-job training needs be carried out by care managers in collaboration with the Government.
- Awarding incentives is likely to attract relatively educated volunteer caregivers. This will possibly raise the caregivers’ productivity, care morale and increased spirit of volunteerism.
  Batswana are people who are known to respect and maintain their culture, especially the culture of peace and coexistence, and traditionally working together for the benefit of one another or “letsema” and caring for one another. Batswana are traditionally known for displaying the virtue of *molaletsa* or collaboration in activities such as building one another’s house or kraals.[32,33] The call for caring for one another is deeply enshrined in vision 2016 pillars such as “a just and a caring nation”.[15] Therefore, the feeling by the caregivers that care giving volunteerism finds respect and is of cultural importance explains the strength and vigor of maintaining and keeping up with the process. This has explained the panacea of care giving. However, this spirit of volunteerism has been on the decline as societies continue to abandon traditional and extended family structures in response to modernization, Westernization, eurocentrism, and globalization. The volunteerism spirit, therefore, needs to be revived and bolstered.[2,4,17,25]
- The spirit of volunteerism and caring for one another should be strengthened through continued advocacy and lobbying especially to persons at lower school going age. This will ensure that this culturally rich practice is retained.
  Batswana are also known to be patriotic, peaceful, law-abiding and lovers of their country.[32,33] Having been called upon to assist on care through community home based care program by their government gives weight to the fact that the process of volunteerism has to continue despite challenging environments. The call for the spirit of “*boithaopo*” (volunteerism), “*boineelo*” (service to one another) and “*botho*” (humanity) are deeply grounded in the country’s vision 2016 and are especially guiding principles in HIV/AIDS field of care giving.[2,15] Through the embedded call for volunteerism, Batswana are striving to see the fulfillment of an AIDS free generation through prevention and uplifting their caring tempo, and being mindful of one another’s welfare.[15] This has made care giving a panacea.
  Caregivers alluded to the power of religion as an important factor driving volunteerism. Christianity and most religions of the world are grounded in the principle of helping the less fortunate members of the society, with the Holy Bible leading by example.[34] This drives one to help without any remuneration or expectation. The gospel of Jesus Christ clearly advises the Christians to do well without expectation.[34]
- Batswana should strive to operationalize the caring call enshrined in their vision 2016 document. Leaders at all levels should step up advocacy to the communities.On care giving volunteerism being driven by blood relations and communal responsibility over one another, other studies have found a close match to the Kanye findings that caregivers prefer the clients to be taken care of by families, relatives and community members at home as opposed to hospitals, as they indicated that “blood is thicker than water” (*Kang’ethe paradigm*). This is despite all the challenges associated with carrying out care at homes like lack of adequate care package and social support.[4,10] Studies by Ursula,[35] Sims and Moss[11] and Nurses Association of Botswana,[7] have indicated the wish for clients to be taken care of at home, especially during their last days and possibly meet their death in the hands of the loved ones. In Kanye, however, besides blood relations and communal responsibility for one another, the wish to have clients taken care of at home is attributable to the nonconductive environment that the clients are subjected to in the referral hospital, making communities to opt to take care of their sick communities at home. The Kanye SDA referral hospital was found to challenge both the clients and the caregivers because of its unfriendly state.[4,10]
  Other studies have found that care giving in an African context has been found to be a fulfillment of love and African brotherhood that has empirically been going on for generations.[17] Apparently, this appears to dwindle with time as communities move towards nuclear family set up succumbing to forces of westernization, eurocentricism, modernization and globalization.[4] However, helping one another, caring and being compassionate is one of the pillars envisioned and enshrined in the country’s vision 2016 tenets.[15]
- Communities are encouraged to increase the state of assistance to their distressed members of the society by embracing Vision 2016’s tenet of “being a compassionate and a caring nation”. The vision also espouses and ingrains the call and challenge for care giving volunteerism.[15]

## CONCLUSION

The spirit of volunteerism, without any economic expectation, should be encouraged and embraced by all, especially in care giving. This will make care giving a panacea. This is because most countries of developing world lack adequate resources (human and material) to afford full paid employment in many spheres of social development. It is also a way for communities to learn to shoulder community problems or complement Government assistance. However, the overwhelming nature of HIV/AIDS phenomenon in many developing countries has made care giving perfidious and requires that communities volunteer to fill up the needed scarce human and material resource gaps. But the dynamism of life, especially its economic demands should be respected in that the environment of care giving volunteerism need be conducive and motivational. Giving of incentives, encouragement, motivation and recognition, are all necessary factors to keep the candle and fire of volunteerism burning. This can make palliative care giving a panacea and reduce its state of being perfidious.
